# Pharmaceutical Stability of Romiplostim: In-use Functional Study Using Microscale Thermophoresis New Strategy and Elisa

**DOI:** 10.1007/s11095-026-04085-5

**Published:** 2026-04-23

**Authors:** Jesús Hermosilla, Salvador Casares-Atienza, Julio Ruiz-Travé, Anabel Torrente-López, Antonio Salmerón-García, Jose Cabeza, Natalia Navas

**Affiliations:** 1 Department of Analytical Chemistry, Faculty of Science, University of Granada, Fuentenueva Avenue S/N, 18071 Granada, Spain; 2 Instituto de Investigación Biosanitaria de Granada (Ibs.GRANADA), Av. de Madrid, 15, Beiro, 18012 Granada Spain; 3 Department of Physical Chemistry, Faculty of Science, University of Granada, Fuentenueva Avenue S/N, 18071 Granada, Spain; 4 Department of Clinical Pharmacy, San Cecilio University Hospital, Av. del Conocimiento, S/N, 18007 Granada, Spain; 5 Center for Proteomics and Metabolomics, Leiden University Medical Center, Leiden, The Netherlands

**Keywords:** bioanalytical characterisation, biopharmaceutics, ELISA, functionality, in-use stability, microscale thermophoresis, romiplostim

## Abstract

**Objective:**

Functional stability is crucial for therapeutic proteins at all stages of their lifecycle. Romiplostim (Nplate®) is world-wide used for treating immune thrombocytopenia purpura. Limited in-use stability data on the proper romiplostim handling is publicly available. This study aimed to evaluate romiplostin in-use functional stability focusing on the interaction with its therapeutic target (TPO-R) and to improve the existing functional assessment strategies by developing a new one using microscale thermophoresis (MST).

**Methods:**

Reconstituted romiplostim (Nplate®) samples were analysed. MST and ELISA functional methods were developed and optimized ad hoc to assess romiplostim functional stability. Far-UV-Circular-Dichroism and Intrinsic-Tryptophan-Fluorescence-Spectroscopy were used to ensure MST results by confirming the conformational stability of labelled TPO-R. In-use stability was assessed by subjecting romiplostim to conditions related to hospital handling, e.g. agitation and exposure to natural light; also, freezing and artificial light irradiation were checked as forced degraded condition.

**Results:**

MST and ELISA were described and validated by their figures of merit. Regarding romiplostim functional stability, both approaches yielded similar stability profiles, but MST detected subtle functional declines that were not revealed by ELISA. Furthermore, MST revealed insights into romiplostim binding stoichiometry, suggesting 1:2 interaction at high concentrations.

**Conclusion:**

Regarding functionality, romiplostim was particularly sensitive to light exposure, with less sensitivity to agitation and freezing. These findings also demonstrate that MST serves as new orthogonal strategy to ELISA for assessing functionality, even providing additional data on binding dynamics and stoichiometry. This new MST strategy is applicable for any type of functional study of therapeutic proteins.

**Supplementary Information:**

The online version contains supplementary material available at 10.1007/s11095-026-04085-5.

## Introduction

Romiplostim is a therapeutic Fc-fusion peptibody formulated under the medicine name Nplate® (Amgen Europe B.V, Breda, The Netherlands). It is indicated for the treatment of adult chronic immune (idiopathic) thrombocytopenic purpura (ITP) in splenectomised patients who are refractory to other treatments (e.g., corticosteroids, immunoglobulins) [1]. Structurally, it is a homodimer composed of two identical repeats of a construct IgG1 Fc fused by the C-terminal to two thrombopoietin receptor (hTPO-R) agonist peptides separated by 8-glycine linkers [2]. It interacts with human Thrombopoietin Receptor (hTPO-R), also known as myeloproliferative leukaemia protein (c-mpl) with a half maximal Effective Concentration EC50 from 5 to 170 pM [3], stimulates maturation of megakaryocytes and thus, increases the production of platelets. Nplate® is presented as a lyophilised white powder for solution for injection at 500 µg/mL, to be administered once weekly. Its *Summary of Product Characteristics* (SPC) [1] indicates a stability after reconstitution of 24 h stored at 2–8 ºC and 24 h stored at 25 ºC, when protected from light and kept in the original vial. In addition, stability data beyond expiry dates or in-use stability data are limited, making it necessary to conduct in-depth stability studies to gain insight into degradation routes and sensitivity to different conditions/practises.

Among all the critical quality attributes, the interaction of a therapeutic protein with its target is crucial to achieve a desired therapeutic effect. Biological activity is defined by the EMA as “the specific ability or capacity of a product to achieve a defined biological effect”, and is addressed by animal-based, cell-based and biochemical assays and other procedures such as ligand and receptor binding assays [4]. Enzyme Linked Immunosorbent Assay (ELISA), and Surface Plasmon Resonance (SPR) are the main recommended methods by EMA and regulatory agencies to assess biological activity, that is, the functionality. Several techniques are aimed at measuring and quantifying the affinity of biomolecular interactions, being the immobilisation free binding assays essential since they provide more relevant conditions regarding *in vivo* interactions.

MicroScale Thermophoresis (MST) is a growing modern biophysical technique used to quantify the interaction of biomolecules [5]. To do so, one of the binding partners is conjugated to a fluorescent probe, whereas the other binding partner remains without any labelling. MST instruments detect fluorescence changes over time caused by the migration of molecules upon a microscopic temperature gradient induced by an infrared laser, which varies depending on several structural aspects like molecular size, hydration or charge. MST stands out for its low sample consumption, sensitivity, simplicity and low instrument maintenance, allowing for the determination of equilibrium dissociation constants (K_D_) in the low picomolar range [6]. MST is widely used to study interactions of peptides and proteins [7], as well as small molecules during drug discovery [8] and pharmaceutical development stages [9]. Recently, a universal biosensing strategy based on DNAzyme MST has been proposed for sensitive target detections [10]. As MST allows for the quantitative analysis of protein–protein interactions (PPIs), it can be very useful to study the interaction of monoclonal antibodies (mAbs) with their therapeutic targets. However, scientific literature dealing with mAbs interactions by MST is scarce [11, 12]. The interaction of TNFα with the therapeutic mAb adalimumab and the Fc fusion protein etanercept was assessed for the first time by MST, determining biphasic binding curves with low and high *K*_*D*_ values [11]. In a different context, MST was applied to study the interaction between amyloid β-peptides (Aβ) monomers (Aβ42) and three commercially available mAbs directed against Aβ, which play a major role in the pathogenesis of Alzheimer’s disease, with the aim of determining the antigen–antibody complex formation and binding affinities [12].

In this research, for the first time, the functional stability of romiplostim (Nplate®), intended as its capacity to bind to its target (the TPO-R), is evaluated using MST by determining the affinity of such peptibody for the TPO-R, its therapeutic target. Specifically, two orthogonal functional methods were selected to assess such functional stability in this work: a biochemical strategy using an indirect ELISA and the biophysical approach using MST. These methods were properly ad hoc developed and validated from an analytical point of view for further assessing the in-use functional stability of the reconstituted medicine. To do that, a selection of relevant environmental stress factors that the medicine is likely to encounter during handling/storage in hospitals were selected and the medicine was subjected to them before analysis to evaluate romiplostim degradation. It is well known that protein-based biotherapeutics can suffer degradation due to routine handling or unintentional mishandling, leading to unnoticed physicochemical changes that can compromise the clinical safety and efficacy of the product [13]. Mishandling of these bioproducts can be related to their storage conditions. Therefore, the stress factors selected and included in this study are agitation, exposure to natural light, freezing and exposure to light irradiation in an aging chamber, all of them performed for 24 h. These tests offer valuable new insights into the functional stability of this peptibody, aiding in the assessment of potential consequences associated with its routine handling in hospital settings, particularly concerning its functionality. In addition, this work provides two new protocols to quantify romiplostim functionality, one using ELISA and the other using MST, demonstrating this last for the first time its usefulness as new strategy to check functionality in therapeutics mAb-derived product, comparing also the results by the traditional ELISA method.

## Materials and Methods

### Romiplostim (Nplate)

Nplate (Amgen Europe B.V, Breda, The Netherlands) was used for this study. Vials containing 500 µg of romiplostim were reconstituted in 0.5 mL water for injection to give a final concentration of 500 µg/mL. The list of excipients includes mannitol (E421), sucrose, L-histidine and hydrochloric acid (for pH adjustment). Romiplostim is a recombinant non-glycosylated Fc-peptide 59 KDa fusion protein (peptibody) produced in E. coli [1].

### Physicochemical Analytical Methods

#### Far UV Circular Dichroism (CD) Spectroscopy

CD spectroscopy in the far UV region (190–260 nm) was applied to assess the secondary structure of romiplostim. Romiplostim was analysed at the medicine concentration of 500 mcg/mL and spectra were recorded using a Jasco J-815 CD spectropolarimeter (Tokyo, Japan) equipped with a Peltier system for temperature control (25 ºC), every 0.2 nm with a scan speed of 100 nm/min. A total of 20 accumulations were registered using a quartz cuvette with a path length of 0.2 mm. The blank (Nplate® excipients without the protein) was measured and then subtracted from the samples. Finally, means-movement smoothing was applied to all the spectra with Spectra Analysis software (Jasco, Tokyo, Japan). Secondary structure content was estimated by Dichroweb online server [14] using the analysis programme CDSSTR analysis and selecting the proteins dataset 4 [15] in addition to the usual CD spectral features.

#### Intrinsic Tryptophan Fluorescence Spectroscopy (IT-FS)

IT-F of unlabelled thrombopoietin receptor (TPO-R) and labelled TPO-R (*l*-TPO-R) was assessed to detect changes in tertiary structure upon labelling. Emission fluorescence spectra were recorded between 310 and 400 nm by selectively exciting tryptophan residues at 298 nm, using a Cary Eclipse spectrofluorometer from Agilent Technologies Inc. (Santa Clara, CA, USA) equipped with a Peltier system for temperature control (25 ºC). Spectra were recorded at a scan speed of 600 nm/min and a total of 3 replicates were averaged per sample. Spectral centre of mass was calculated in that range using Eq. 1 to evaluate conformational modifications.

1 $$\mathrm{C}.\mathrm{M}=\frac{\sum_{\mathrm{i}=1}^{\mathrm{n}}({\mathrm{f}}_{\mathrm{i}}{\uplambda }_{\mathrm{i}})}{\sum_{\mathrm{i}=1}^{\mathrm{n}}{\mathrm{f}}_{\mathrm{i}}}$$

### Functional Bioanalytical Methods

#### Enzyme Linked Immunosorbent Assay (ELISA)

Functionality was assessed by means of the biological activity quantification measured by an indirect and non-competitive ELISA method, ad hoc developed and validated. The optimization of the method was focused on the determination of the better conditions for the TPO-R/romiplostim reaction and for the conjugate reaction. In brief, Maxisorp immune plates were sensitised by adding 100 µL/well of 0.5 µg/mL of TPO-R diluted in 0.1 M carbonate buffer solution pH 9.6 (prepared with sodium carbonate (Panreac, Barcelona, Spain) and sodium bicarbonate (Panreac, Barcelona, Spain)) and incubated overnight (18 h) at 4ºC. The plates were washed four times with 200 µL/well of PBS-Tween 20 pH 7.4 containing 0.3% (v/v) Tween 20 (Guinama, Valencia, Spain); PBS was prepared using sodium chloride, potassium chloride, disodium phosphate monohydrate and potassium phosphate monobasic (Panreac, Barcelona, Spain). To block wells and eliminate nonspecific absorptions, 200 µL/well of blocking buffer (PBS-Tween 20 pH 7.4 containing skimmed milk 2% (w/v)) were added and the plates were incubated for 2 h at 37ºC. After that, wells were washed four more times and filled with 100 µL per well of increasing concentrations of romiplostim, i.e. 0.1 µg/mL, 0.25 µg/mL, 0.5 µg/mL, 1 µg/mL, 5 µg/mL and 10 µg/mL, diluted in 0.1 M carbonate buffer pH 9.6; each concentration was tested in triplicate and plates incubated 45 min at 37ºC. After that, plates were washed four times with PBS-Tween 20 pH 7.4 and incubated 37ºC for 30 min with 100 µL per well of secondary anti-Human antibody IgG1 Fc conjugated with peroxidase, HRP (Millipore, Temecula, USA) diluted 1:10,000 in 0.1 M carbonate buffer pH 9.6. Next to wash four times again, 100 µL of the substrate solution, i.e. O-Phenylenediamine Dihydrochloride (OPD) (Sigma Aldrich, Madrid, Spain) was added per well and incubated 20 min in darkness at room temperature. To stop the reaction, 50 µL of 1 M H_2_SO_4_ (Panreac, Barcelona, Spain) were added per well. Absorbance was recorded at 450 nm and 620 nm, and the analytical signal was the difference between the two values, acquired by a TECAN SUNRISE™ microplate absorbance reader for 96-well plates connected to a computer loaded with XFluor4 software (Tecan, Austria, GMBH). All incubations at 37 ºC were performed in a universal digitronic precision oven P Selecta® (J.P. Selecta, s.a. Abrera, Barcelona, Spain).

Validation of the method was performed in terms of calibration model, precision and accuracy in accordance with the ICH Q2(R2). Due to the impossibility of getting proper romiplostim standard samples, reconstituted unstressed samples of Nplate® (500 µg, romiplostim) analysed within the 24 h stability period indicated by the SPC were considered as such standard for ELISA method validation. To select the calibration model, romiplostim standard solutions were prepared at 0.25, 0.5, 1, 5 and 10 µg/mL by diluting the romiplostim solution of 500 µg in 0.1 M carbonate buffer pH 9.6. Each standard concentration was prepared and analysed sixfold. The working concentration interval was set between 0.5 and 10 µg/mL romiplostim, with a target concentration of 5 µg/mL (placed in the middle of the interval). The precision and accuracy were evaluated at these three concentrations. The precision was tested as repeatability (intraday precision) and intermediate precision (interday precision considering two consecutive weeks). For repeatability study, nine standard solutions were prepared the same day and under the same experimental conditions: 3 samples at 0.5 µg/mL, 3 samples at 5 µg/mL and 3 samples at 10 µg/mL by diluting the romiplostim solution of 500 µg in 0.1 M carbonate buffer pH 9.6. For evaluating the intermediate precision, the three standard solutions at 0.5, 5 and 10 µg/mL were freshly prepared on 2 different weeks and under the same experimental conditions. Both repeatability and intermediate precision were determined as the relative standard deviation (RSD, %) of the concentrations estimated using the standard calibration curve. Accuracy was also estimated along the concentration interval of working, therefore, standard solutions of 0.5, 5 and 10 µg/mL romiplostim were prepared by diluting the romiplostim solution of 500 µg in 0.1 M carbonate buffer pH 9.6 and analysed; after that, the mean recovery percentage (R, %) was determined.

This ELISA method was also used to evaluate whereas the fluorescence labelling of TPO-R required for the MST method affected the interaction with romiplostim. For this purpose, the romiplostim concentration checked were 0.1, 0.25, 0.5, 1.0, 5.0, 10.0 and 15.0 µg/mL. In this case, EC50 values in nM were derived from the sigmoidal curves obtained using GraphPad Prism software version 7.

#### Microscale Thermophoresis (MST)

MST was selected as an orthogonal functional methodology to ELISA for assessing the biological activity of romiplostim, based on tracking the romiplostim/TPO-R interaction in solution by means of the experimental calculation of the dissociation constant (K_D_). Therefore, a method was developed for such purpose. In brief, 50 µg of recombinant human thrombopoietin receptor, carrier free (TPO-R) from Bio-Techne Ltd (Abingdon, UK) were reconstituted in sterile 150 mM PBS pH 7 and centrifuged 15 min at 14,000 rpm in a MIKRO 220R centrifuge (Hettich, Tuttlingen, Germany). For labelling, Protein Labelling Kit RED-NHS 2nd Generation from NanoTemper Technologies GmbH (Munich, Germany) was used. The labelled stock solution was aliquoted in fractions that were flash frozen in liquid nitrogen and stored at −80ºC. To obtain optimal dose–response curves, several MST parameters were optimised: target (*l*-TPO-R) concentration, ligand (romiplostim) range concentrations and MST signal. Target and ligand samples were centrifuged 15 min at 14,000 rpm prior to the MST analyses to eliminate any possible aggregation. Protein concentration was prior measured by UV spectroscopy at 280 nm using a Varian Cary 50 Bio UV–Vis Spectrophotometer, Agilent Technologies Inc. (Santa Clara, CA, USA). 20 nM was selected as the target concentration whereas the ligand maximum and minimum assay concentrations were 100 nM and 3 pM respectively. Therefore, serial dilutions 1:1 with the assay buffer, which consisted of 150 mM PBS pH 7 with 0.05% Tween 20, were prepared following software (*MO. Control version 1.6.1*) instructions. Premium capillaries (NanoTemper, Germany) were used for loading the samples in the Premium Capillary Tray Monolith NT.115 Series (NanoTemper, Germany), a total of 16 capillaries were loaded onto the tray. Monolith NT.115 instrument (NanoTemper, Germany) parameters were set as follows: temperature at 25 ºC, excitation power at automatic and MST power at high. Data treatment was done by the *MO. Affinity Analysis software v2.3*. Each romiplostim sample was analysed in triplicate and averaged. A K_D_ model was selected for all the analyses. Target concentration was fixed in each analysis to obtain the best setting parameters. The MST analysis was done at 1.5 s for all samples to confirm binding and subsequently customised manually to obtain curves with the highest signal to noise ratios. Capillaries in which fluorescence intensity was out of the ± 20% threshold were discarded to guarantee homogeneity of initial fluorescence as well as capillaries with presence of aggregates. Results are expressed as average K_D_ mean ± confidence (IC, 68%).

### In-use Stability Study

Romiplostim (Nplate®, 500 µg/mL) was tested for its functional stability upon manipulation evaluating the biological activity. For this purpose, several stress factors were selected and applied to solutions of the medicine once reconstituted. Firstly, the product was aseptically reconstituted in the Pharmacy Unit of the San Cecilio University Hospital (Granada, Spain) and stored refrigerated (2–8ºC) until analysis. Aliquots (120 µL) of the medicine solution were placed in amber/clear 2 mL vials and subjected to the following conditions: horizontal agitation at 300 rpm on a mechanical laboratory shaker (Model 3006, Gesellschaft für Labortechnik, Burgwedel, Germany) at room temperature with thermal excursions between 24–32ºC and protected from light during 24 h; exposition to day light through the glass of a window at room temperature with thermal excursions between 24–32ºC during 24 h; storage inside a freezer at −20ºC protected from light during 24 h. A more intense forced degradation test was also conducted by exposing romiplostim aliquots to accelerated light irradiation of 250 W/m^2^ during 24 h in a Solarbox 3000e RH aging chamber (Cofomegra, Milan, Italy). This was used as positive control for degradation.

Romiplostim unstressed samples analysed within the 24 h stability period indicated by the SPC were considered the control samples for comparison purposes. The functional analysis of the stressed samples was performed by simultaneously analysing control and stressed romiplostim samples. The results were statistically compared using the student’s *t* analysis with 95% of confidence to confirm whether the ability of romiplostim to bind to its target TPO-R had been altered. For this purpose, the biological activity (expressed as % of remaining activity) obtained by ELISA or the K_D_ obtained by MST were compared to the values of control samples. The reported biological activity was the average value obtained at the three target concentrations of the ELISA method (0.5, 5 and 10 µg/mL), whereas K_D_ value was the average of three instrumental replicates.

## Results and Discussion

### Enzyme-Linked Immunosorbent Assay (ELISA)

#### Optimisation and Validation

The indirect and non-competitive ELISA method was ad hoc developed and validated for evaluating the biological activity of romiplostim (by measuring its specific interaction with TPO-R, functionality), and for comparing the interaction of TPO-R with romiplostim before and after chemical labelling, to check the viability of using MST, where a fluorescent label is required. The method was based on previous own research studies [16–19] and available literature [20]. Then, the optimization focused mainly on the TPO-R/romiplostim concentration ratio, since in the step of revealing this union by binding to the conjugate, we adjusted the experimental conditions from our previously developed ELISA methods. Once all these conditions were selected (ELISA section in *Materials and Methods*), validation started from the selection of the calibration model and the concentration interval of working. Then, it was selected the mathematical function that best fitted the experimental data. After testing several mathematical models, a logarithmic one was selected as the most suitable for calibration purposes, based on the coefficient of determination R^2^ (99.44%), which demonstrated the goodness of fitting (curve shown in Supplementary Fig. 1). Considering this graphical distribution (absorbance* vs *µg/mL romiplostim), the selected optimum concentration for the analysis of romiplostim (working interval) were between 0.5 and 10 µg/mL, with the target concentration of 5 µg/mL (placed in the middle of the interval). Therefore, the repeatability and intermediate precision -both expressed as RSD (%)-, and the accuracy -expressed as recovery- were studied at these three concentrations. Results are shown in Table I. For immunoassays, it is recommended to adhere to minimal acceptance limits of 20%, which indicates that the model met the precision criteria specified for bioanalytical method validation [21, 22]; to highlight that except for the lower concentration (0.5 µg/mL), the RSD (%) was lower than 5%, indicating good precision of the ELISA method at these concentrations (10 and 5 µg/mL). Recovery values ranged from 99 to 127%, confirming satisfactory quality of the ELISA method proposed for evaluating the biological activity romiplostim. This is particularly noteworthy given the substantial variability inherent in immunoassay-based methods [18].

**Table I:** Accuracy and Precision of the ELISA Based Method for Romiplostim

Romiplostim Concentration (µg/mL)	R (%)^a^	RSD (%)^b^	
		Intra-day	Inter-day (2 weeks)
10	108.0	4.5	4.6
5	126.9	1.1	1.2
0.5	99.4	9.8	19.6

^a^Recovery value based on three determinations; ^b^Relative standard deviation based on three (intraday) and six (Interday) determinations

#### In-use Stability Study: ELISA

Samples of reconstituted romiplostim were subjected to several stress factors that mimic extreme conditions to which the drug could be exposed when handling before administration to patients, i.e. agitation, exposition to day light, thermal excursion, accelerated exposition to light, and freezing/thawing. The stress specific conditions are described in section “In-use Stability study in *Materials and Methods*”.

**Fig. 1:**
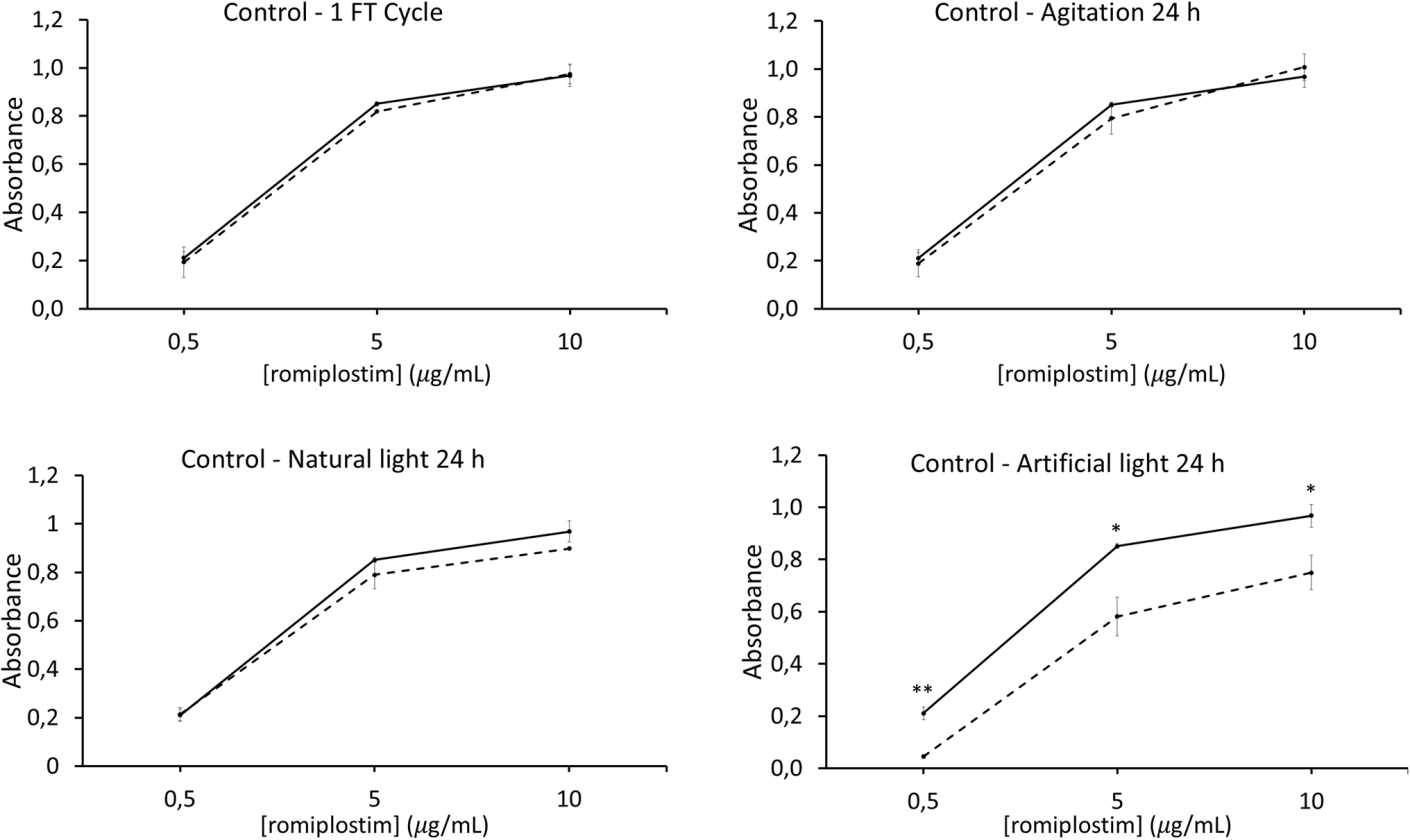
Functional stability study: ELISA binding assay (0.5, 5 and 10 µg/mL) for the stressed samples (dashed line) compared to control samples (solid line) of romiplostim (*: *p*-value < 0.05; **:* p*-value < 0.001).

After subjecting the romiplostim samples to these stress factors, they were analysed to obtain the ELISA calibration graphs, which were compared to the control ones, to determine whereas the stressor applied promoted a decrease in the binding capacity of romiplostim to its therapeutic target, the TPO-R. With that aim, a student’s *t* analysis was performed to compare the average values from different groups. In Fig. 1, data points marked with asterisks indicate significant (one asterisk, *p*-value < 0.05) and very significant (two asterisks, *p*-value < 0.001) differences among stressed samples and controls. It can be seen clearly that accelerated light exposure during 24 h provokes a deep decay in romiplostim binding capacity to TPO-R since significantly lower absorbance values were observed with respect to the controls. It is well known that oxidations are produced in therapeutic proteins when subjected to accelerated light exposition [23], affecting binding capacity if degradation occurs in the protein binding site. On the contrary, no significant differences were found in the other conditions, including exposition to day light, then indicating that the stressors applied did not alter romiplostim/TPO-R interaction. The estimated remaining biological activities of the romiplostim stressed samples, expressed in %, are shown in Table II, which are indistinguishable from the controls, as indicated by the statistics test applied.

**Table II:** Estimation of the Remaining Biological Activity in Stressed Samples of Romiplostim by ELISA

Stress Condition	Remaining Activity (%)
1 F-T Cycle	94.7 ± 7.6
Natural light 24 h	84.8 ± 14.0
Agitation 24 h	96.2 ± 19.3
Artificial light 24 h	42.3 ± 8.8

### Microscale Thermophoresis (MST)

#### MST Method Development

**Fig. 2:**
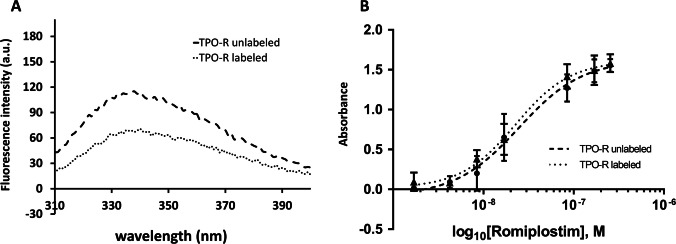
(**A**) Emission fluorescence spectra of TPO-R before (dashed line) and after chemical labelling (dotted line) with RED-NHS 2nd Generation dye; excitation was selected at 298 nm. (**B**) Dose–response curves of romiplostim as titrant against TPO-R obtained by an indirect ELISA. Absorbance is represented as a function of romiplostim concentration in molar (M) units.

Firstly, several experiments were carried out to determine if MST strategy was adequate for evaluating the biological activity of romiplostim. With that aim, studies using IT-FS and ELISA were performed to conclude whether the tertiary structure and the functionality of TPO-R were compromised or not upon labelling with the RED-NHS 2nd Generation dye required to analyse romiplostim/TPO-R interaction by MST. TPO-R emission IT-F spectra showed a spectral C.M. of 349 nm, whereas *l*-TPO-R C.M value was 350 nm, indicating that tryptophan environment (indicator of the tertiary structure) was not significantly altered, although a significant decay of the fluorescence signal was observed (Fig. 2A). Besides, the developed and validated ELISA method was used to compare the romiplostim/TPO-R interaction before and after labelling. A dose–response curve was obtained for both TPO-R and *l*-TPO-R as a function of romiplostim concentration. In both cases saturation was achieved, and EC50 parameters were also calculated by fitting data to a sigmoidal non-linear regression; these values were 24.7 nM and 22.7 nM for TPO-R and *l-*TPO-R, respectively. Therefore, it was demonstrated that *l*-TPO-R maintained its functionality (Fig. 2B). Gathering the results of these experiments, it could be concluded that the labelling strategy was adequate since TPO-R conformation and functionality were not altered. Decay of the fluorescence signal could be attributed to a quenching effect promoted by binding of the dye. MST strategy was therefore proposed to assess the biological activity of romiplostim.

**Fig. 3:**
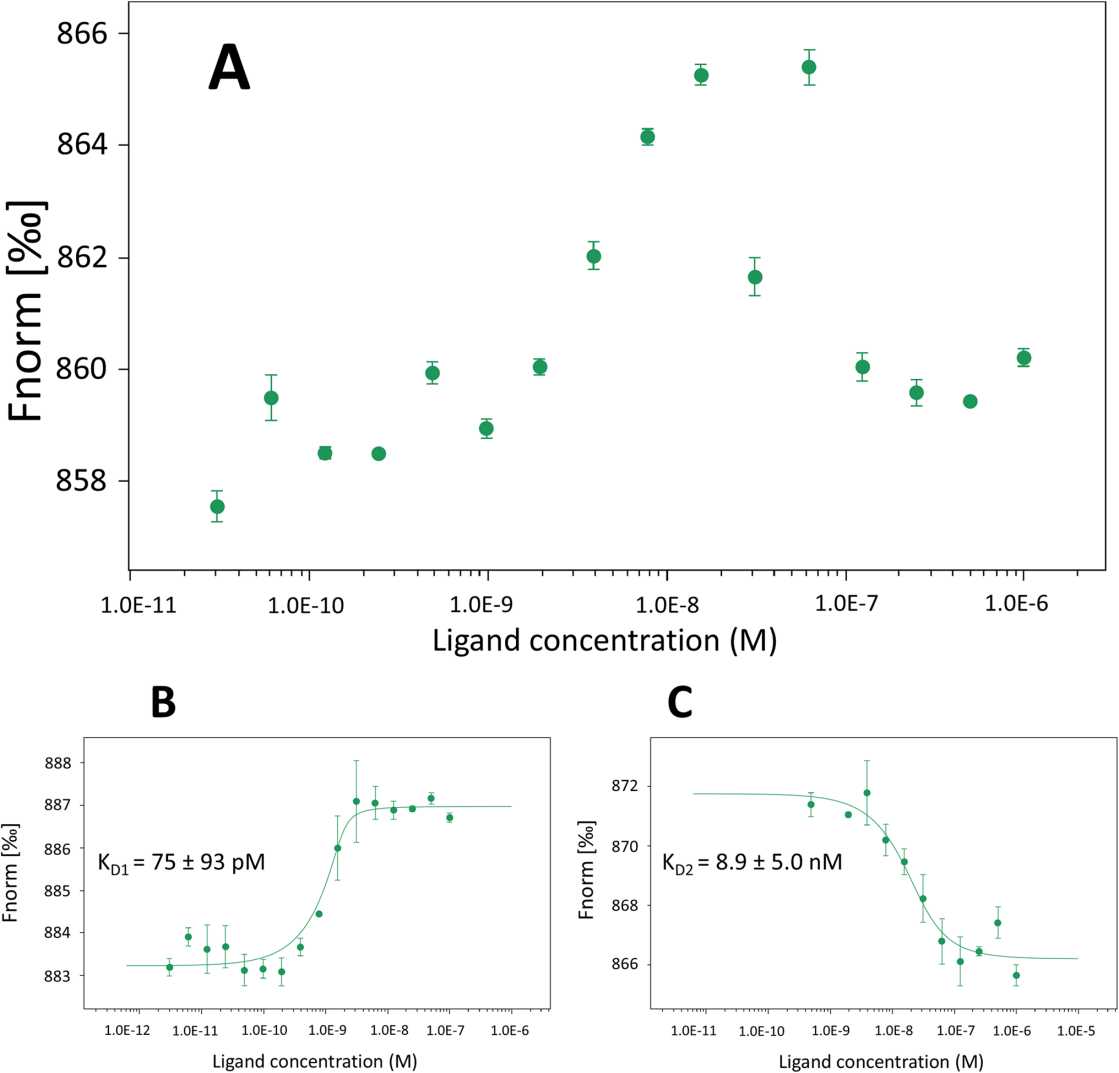
Functional characterisation of the interaction of romiplostim with *l*-TPO-R by MST. In (**A**), a biphasic behaviour was confirmed. In (**B**) and (**C**), both equilibria were analysed independently; romiplostim first equilibrium was characterised by a K_D_ of 75 ± 93 pM, whereas the second equilibrium was characterised by a K_D_ of 8.9 ± 5.0 nM.

Considering the results obtained by the ELISA method, MST experiments were started by setting the maximum ligand (romiplostim) concentration at 1 µM and performing serial dilutions (1:1) in PBST 0.05%, fixing target concentration at 20 nM and MST power at high. A sigmoidal curve was detected in the range 3.05 × 10^−11^ M-6.25 × 10^–8^ M, confirming binding in this region (Fig. 3A). A decrease in the Fnorm values was observed at concentrations above 6.25 × 10^–8^ M (Fig. 3A), this might indicate a second equilibrium. To confirm this, two additional independent experiments were carried out by adjusting the concentration interval and analysis times of the MST traces, since the K_D_ model implemented in *MO. Affinity Analysis software v2.3* only allows for a 1:1 stoichiometry equilibrium characterization. The first equilibrium was characterised by a K_D1_ of 75 ± 93 pM (Fig. 3B), whereas the second equilibrium by a K_D2_ of 8.9 ± 5.0 nM (Fig. 3C). This approach was feasible because both obtained K_D_ values differ by 3 orders of magnitude Here, we focused on the study of the high affinity interaction, i.e., that with the lower K_D_ value; therefore, the maximum ligand concentration was lowered to 100 nM, confirming binding in the range of 3 pM to 100 nM (Fig. 3B). Once binding was confirmed, several experiments by lowering target concentration were conducted to try minimising the use of *l*-TPO-R: 5 nM, 10 nM and 20 nM. However, 20 nM was needed since no amplitude in the signal between the bound and unbound states was detected for the other concentrations, i.e., 5 and 10 nM (Supplementary Data Fig. 2). After that, the relative fluorescence of the experiments was analysed at different times: 1.5 s, 2.5 s, 5 s and 10 s. All the experiments were analysed within the interval 1.5–2.5 s, as it was the region in which the S/N ratio of the sigmoid curve was higher (Supplementary Data Fig. 2). Finally, a set of experiments was also conducted lowering the MST signal to medium; nevertheless, high MST signal was again needed for the higher signal to noise ratio of the interaction (Data not shown). For the second equilibrium (Fig. 3C), MST traces were analysed at 5 s for the highest S/N ratio observed. This biphasic behaviour has been previously characterized for other biotherapeutics [11], as Contreras *et al*. previously discussed for adalimumab and etanercept, suggesting a 1:2 stoichiometry; this can be explained in our case by the multiple valence of romiplostim, in which four TPO analogue synthetic peptides are linked to a IgG1 Fc backbone [3]. An asymmetric arrangement of peptides within the romiplostim structure [2] might be the cause of the existence of a second equilibrium with a lower affinity.

#### In Use Stability Study: MST

**Fig. 4:**
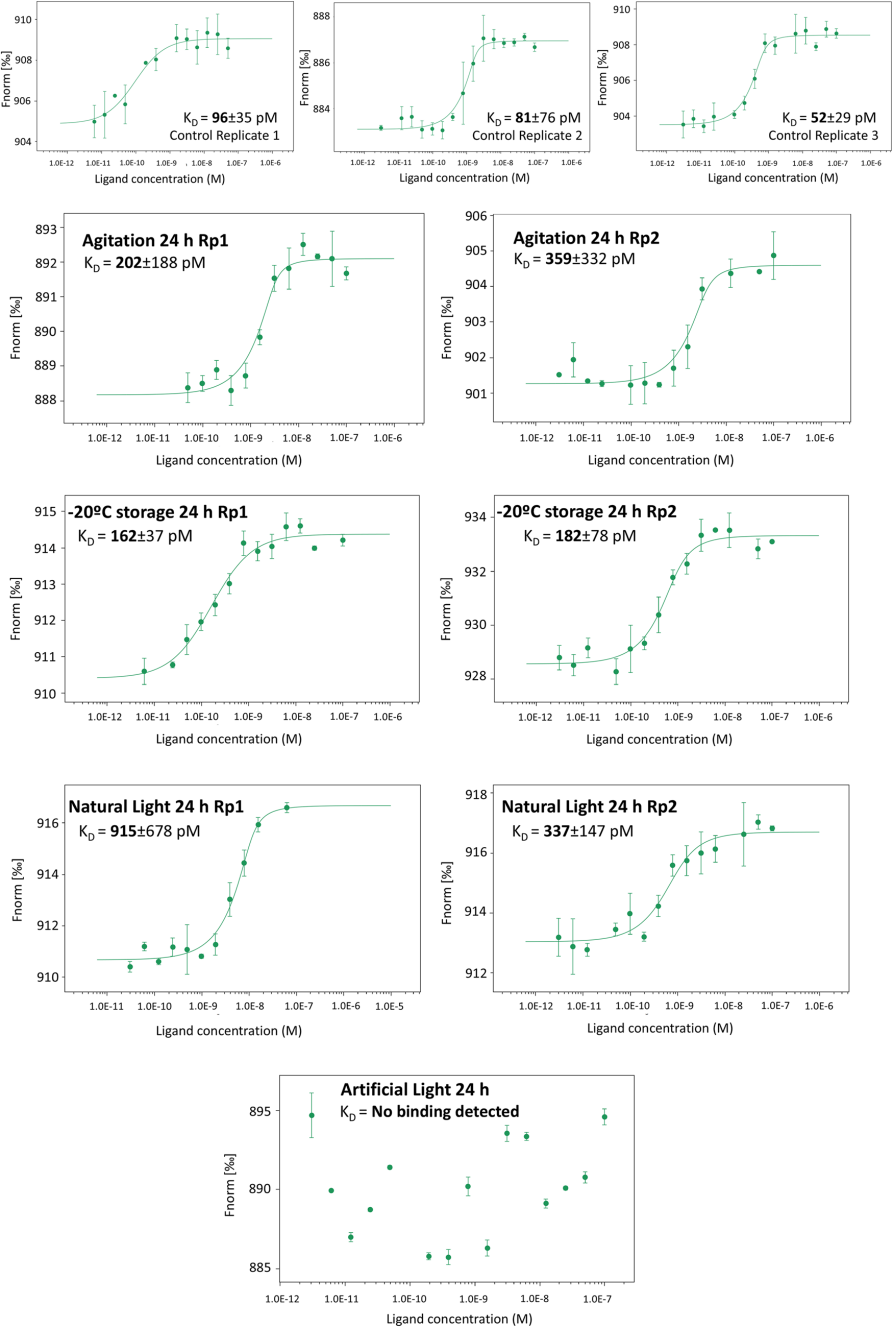
Functional stability study: MST dose–response curves representing normalized fluorescence (%) against ligand (romiplostim) concentration for three romiplostim control samples and two samples for the condition tested. No dissociation constant was derived when romiplostim was subjected to artificial light regardless of the analysis time of the traces (1 to 20 s). Dissociation constants are expressed as K_D_ confidence [pM].

Romiplostim/*l*-TPO-R interaction was assessed by MST using several samples of romiplostim. Specifically, three independent medicine unit samples were analysed to estimate their respective K_D_ values (Fig. 4). The K_D_ values determined were 96 pM, 81 pM and 52 pM for replicates 1, 2 and 3 respectively. These values are in good agreement with those reported in a recent study [24]. In the mentioned study, the researchers conducted a biosimilar/originator (Nplate®) physicochemical and biological comparison and determined the K_D_ of both biotherapeutics by Bio-Layer Interferometry. They estimated the K_D_ of romiplostim (Nplate®) in the picomolar range: 88 pM [39–140] pM. On the other hand, our K_D_ values estimated by MST were lower than those found in published literature: 0.51 nM [25], and those disclosed in the CHMP assessment report for Nplate® [26], which cover a wider range (0.51–14 nM); K_D_ was in this case determined by surface plasmon resonance (SPR), indicating that this parameter varies depending on the determination method. SPR is the preferred method to detect hight affinity interactions [6], although it has come some disadvantages associated to the fact that it requires immobilisation of one of the partners, thus changing binding kinetics and eventually overestimating the dissociation constant of the interaction [27]. Romiplostim interacts with *l*-TPO-R with a very high affinity, maintaining a great capacity to saturate *l*-TPO-R binding site despite the stress conditions reproduced in this study. Nevertheless, differences were found in romiplostim affinity when stressors were applied. In Fig. 4, representative dose–response curves for all conditions are shown: 2 samples were studied for every stress condition selected.

In the case of romiplostim samples subjected to horizontal agitation at 300 rpm during 24 h (RT), the K_D_ values determined were 202 pM and 359 pM, values that increased with respect to control samples, although significant differences could not be confirmed due to their high standard deviation values (Fig. 4); this was attributed to the less defined sigmoidal curve, with fewer central points. It is noteworthy to point out that the samples were also influenced by temperature excursions that varied from 24 ºC to 32 ºC. Agitation of biotechnological medicine solutions is a rutinary clinical practice to ensure total reconstitution of the active ingredient, it also occurs during all the stages from development to patient administration, as it can occur during transportation of the medicine dilutions for administration. A vigorous shaking might be detrimental to the functional stability of biotherapeutics as it might trigger the formation of high molecular weight aggregates or particulates, particularly when proteins are exposed to air/liquid interfaces [28]. Romiplostim functionality was clearly affected by this condition, although it maintained a great activity despite the prolonged time of the test; this situation that can become realistic during transportation from healthcare centres to homes.

A clear increase in the K_D_ parameter was observed in samples undergoing one freeze/thaw cycle; the two samples analysed had K_D_ values of 162 pM and 182 pM. Data dispersion was much lower in this case (Fig. 4), and the sigmoidal curves showed a more binding regime behaviour with more central concentration points defining the curve [29]. Freezing therapeutic proteins is usually discouraged by the biopharmaceutical companies; in this case, the manufacturer Amgen Inc. indicates not to freeze the medicine once reconstituted. This is, however, a common mishandling concern that might occur accidentally during home-delivery [30], or at some locations inside refrigerators, rendering the biopharmaceutic product useless. Numerous degradation routes can be triggered by freezing–thawing proteins, such as partial unfolding of protein molecules that could result in protein aggregation [31], cold denaturation [32], ice formation, changes in solute concentration due to water crystallization, eutectic crystallization of buffer solutes, and resulting pH alterations [33, 34]. In this work, two romiplostim samples were frozen (−20ºC) for 24 h and thawed at room temperature to be analysed by MST, concluding that the medicine functionality was affected by this condition even though samples underwent only a freeze/thaw cycle.

**Fig. 5:**
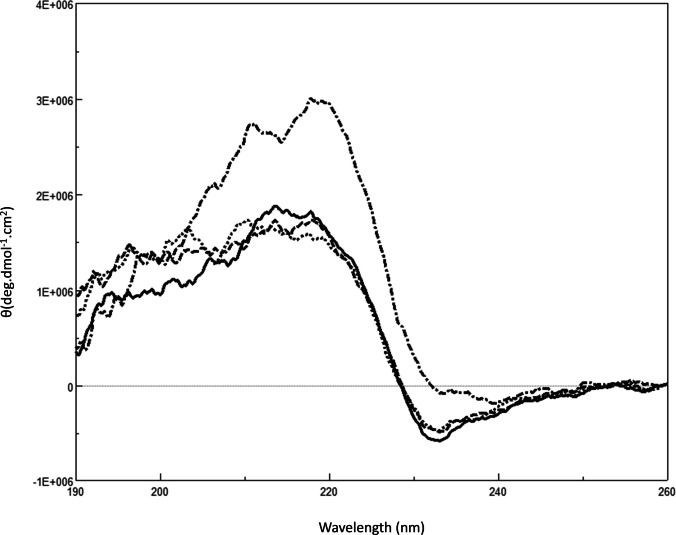
Far UV CD spectra of romiplostim samples subjected to agitation for 24 h (dashed line); natural light for 24 h (dotted line); artificial light for 24 h (dash-dotted line) and control (solid line). Ellipticity, θ (deg.dmol^−1^.cm.^2^) is represented as a function of wavelength (nm).

The condition that showed the highest impact on K_D_ was the exposition to natural daylight, both samples were characterised by K_D_ values of 915 pM and 337 pM. Natural light is always present during all the life cycle of a biopharmaceutical product. It is widely known for its deleterious effect on the chemical structure of proteins, leading to oxidation reactions followed by aggregation [23, 35, 36]. In this study, two samples of romiplostim were placed in clear vials near a lab window rendered to the effects of natural light for 24 h. It can be observed a significant increase in the K_D_ values for the two samples analysed, in addition to a clear difference in the values determined for both samples analysed. This variability observed in the MST results could be due to the less reproducible environmental conditions compared to the other stress factors. These experiments were carried out during March–May, so daily irradiance was likely to be different in both experiments. To gain insight into the effects of light, a forced degradation test by subjecting romiplostim to strong artificial light irradiation conditions (250 W/m^2^) for 24 h was conducted [37]. A sample was subjected to this specific condition and no binding was observed, regardless of the analysis time in the MST fluorescence traces (Supplementary Data Fig. 3). In addition, romiplostim secondary structure was assessed by Far UV Circular Dichroism spectroscopy, which revealed a clear modification of romiplostim CD spectrum after subjected to light irradiation (Fig. 5), spectra of samples subjected to agitation and natural light are also shown, which were identical to romiplostim control sample. We conclude therefore that accelerated light exposure induced conformational changes in romiplostim structure with a subsequent loss of binding affinity to its target. Natural daylight exposure for 24 h, on the other hand, creates subtle modifications in romiplostim functionality by MST that is not detected by either CD spectroscopy or ELISA.

### Stability Study: MST *versus* ELISA

**Fig. 6:**
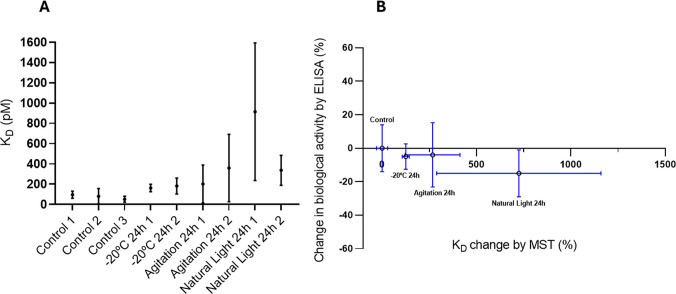
Stability study. MST* versus* ELISA: in A, K_D_ determination for control and stressed samples including replicates, expressed as mean ± SD. In B, correlation graph showing the relationship between activity loss (ELISA) and binding affinity changes (MST) in control and in stressed samples. Y axis shows decay in biological activity (expressed as % of biological activity loss), whereas X axis shows average K_D_ from all replicates (expressed as % increase). A negative lineal correlation indicates a good agreement between both methods. A decay of biological activity signifies a loss of binding affinity of romiplostim.

Romiplostim is a Fc-fusion protein, and as such, it is prone to undergo instability issues due to a variety of physical conditions, i.e., presence of ambient light, temperature excursions, mechanical forces or freeze/thaw cycles, among others. In addition, the medicine (Nplate®) needs prior reconstitution in sterile water for injections, which is a critical step regarding its conformational stability [38]. Romiplostim, the drug candidate in this study, was the subject of several stressors, while two analytical methods had been developed for the analysis of its in-use stability. The first method described is based on an indirect ELISA, a very well-known and standardised technique in which immobilisation of the target protein, i.e. TPO-R, is needed. MST, on the other hand, does not require immobilisation but requires a labelling procedure, which is the most critical aspect when it comes to accurately determining binding affinity, given the possibility of insufficient labelling, protein structure modification or hindering of active sites. To check this, we conducted IT-FS and ELISA studies and concluded that the tertiary structure and function were not affected upon labelling with the dye. Thus, MST methodology has been properly applied to assess the functional stability of romiplostim (Nplate®), obtaining consistent results that were compared to those obtained by ELISA. MST has demonstrated to be a very sensitive and precise approach for affinity measurements, allowing for the determination of not only the dissociation constant (K_D_) -a crucial pharmacodynamic parameter that will ultimately advance its efficacy-, but also the collection of information regarding binding stoichiometry to its therapeutic target (TPO-R). Even though the difference in the thermophoresis signal between bound and unbound states was low for the studied system, as the standard deviation values show for many of the experiments, the method has allowed for the characterisation of several romiplostim control samples, and the K_D_ values determined were in all cases in the range of 50–150 pM. Moreover, the proposed method could detect slight decreases in romiplostim functionality in all the conditions applied to the drug that were not sufficiently registered by the ELISA immunoassay, which detected modification only in the case of submitting romiplostim samples to accelerated light exposition. Figure 6 shows a graph comparing the results of both functional methods. In Fig. 6A, K_D_ values (expressed as mean ± SD) for the control and the stressed samples are shown. In Fig. 6B, each point represents the mean value of biological activity determined by ELISA (Y-axis) and K_D_ determined by MST (X-axis) for each condition. There is a negative linear correlation between the change in biological activity and the change in K_D_ as expected; the loss of biological activity is accompanied by increases in the K_D_ value, which subsequently indicates a loss of affinity with TPO-R. The slight decreases in biological activity (approximately 5%) observed under conditions such as freezing at −20°C or shaking result in increases in K_D_ of 126% for samples stored at −20°C and 280% for shaken samples. For samples affected by natural light, a 15% loss in biological activity results in 724% of K_D_ increase, almost an order of magnitude. As mentioned above, the quantitative nature of the MST assay allows for accurate determination of the K_D_, which is more sensitive detecting modifications in biological activity as here demonstrated.

## Conclusion

In this research, two orthogonal methods based on MST and ELISA have been developed to assess the biological activity of romiplostim (Nplate®). This has allowed the analysis of romiplostim (Nplate®) in-use functional stability upon preparation, being able to draw conclusions about the proper handling of the reconstituted medicine. One of the main pitfalls of the proposed MST method is the necessity for chemical labelling. To prove that this strategy was adequate, the validated ELISA method made it possible to compare the interaction of romiplostim with unlabelled and labelled TPO-R, showing that TPO-R maintained unaffected its capacity to bind to romiplostim. In addition, IT-F data also confirmed that the conformation was maintained after labelling. ELISA and MST methods have yielded similar outcomes since both methods have detected significant decreases of romiplostim functionality after light irradiation stress. Nevertheless, MST strategy has demonstrated to be more sensitive when detecting slight changes in the romiplostim/*l*-TPO-R interaction. MST has proven to be a powerful biophysical method to assess the biological activity of romiplostim, enabling the quantification of the interaction by the determination of the K_D_ parameter in solution at the picomolar range. All in all, romiplostim has proven to be sensitive to all the stress conditions reproduced in this study, particularly to daylight exposure and artificial light exposure. We recommend storing romiplostim protected from light and at constant temperature (2–8ºC).

## Supplementary Information

Below is the link to the electronic supplementary material.

ESM 1 (DOCX 1.06 MB)

## Data Availability

Data will be made available on request.
