# Supplementary material for: Pharmaceutical Stability of Romiplostim: In-use Functional Study Using Microscale Thermophoresis New Strategy and Elisa
Source: Pharm Res. 2026 Apr 23;43(5):1599–612. doi: 10.1007/s11095-026-04085-5 (PMC13269386; doi:10.1007/s11095-026-04085-5)
Supplement: Supplementary file 1 — (DOCX 1.06 MB) [file 11095_2026_4085_MOESM1_ESM.docx]

BIOANALYTICAL METHODS TO ASSESS THE PHARMACEUTICAL STABILITY OF ROMIPLOSTIM: A COMPARATIVE FUNCTIONAL STUDY USING MICROSCALE THERMOPHORESIS AND ELISA.

Jesús Hermosilla^1,2^, Salvador Casares-Atienza^3^, Julio Ruiz-Travé^1^, Anabel Torrente-López^1,2^, Antonio Salmerón-García^2,4^, Jose Cabeza^2,4^, Natalia Navas*^1,2^

^1^Department of Analytical Chemistry, Faculty of Science, University of Granada, 18071 - Granada, Spain.

^2^Instituto de Investigación Biosanitaria de Granada (ibs.GRANADA), Granada, Spain .

^3^Department of Physical Chemistry, Faculty of Science, University of Granada, 18071 - Granada, Spain.

^4^Department of Clinical Pharmacy, San Cecilio University Hospital, 18007 - Granada, Spain.

* Corresponding author

E-mail address: natalia@ugr.es (N. Navas)

Fuentenueva Avenue s/n, 18071 Granada, Spain

Telephone / Fax number: ++ 34 958 242868 / ++ 34 958 243328

| **Supplementary Data Figure 1**. Standard calibration curve for ELISA method.  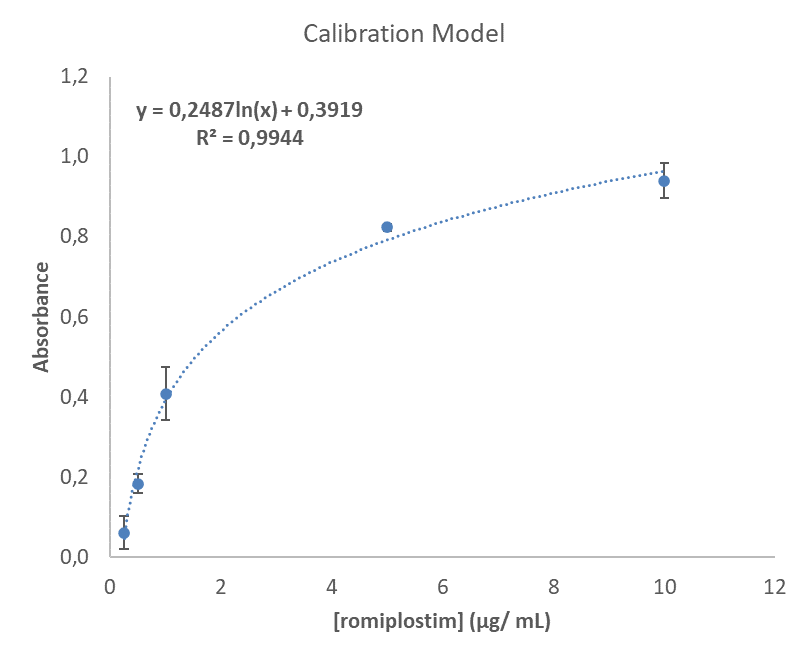 |
| --- |
|  |

**Supplementary Data Figure 2**. Overview of the MST method optimisation. A) A Biphasic behaviour was observed for romiplostim-TPO-R interaction from 1 µM to 30.5 pM of titrant (romiplostim) and 20 nM of target (TPO-R); B) First equilibrium was therefore analysed between 100 nM and 3.05 pM of titrant; target (TPO-R) concentration was lowered to 10 nM (C) and 5 nM (D), observing a significantly lower S/N ratio as compared with B), so 20 nM was selected as target concentration; MST experiments were analysed at different times: 1.5 s (E), 2.5 s (F), 5 s (G) and 10 s (H). It can be observed that sigmoidal behaviour was optimal between 1.5 and 2.5 s, discarding analyses at 5 and 10 s. MST power was in all cases set at high.

| **A)**  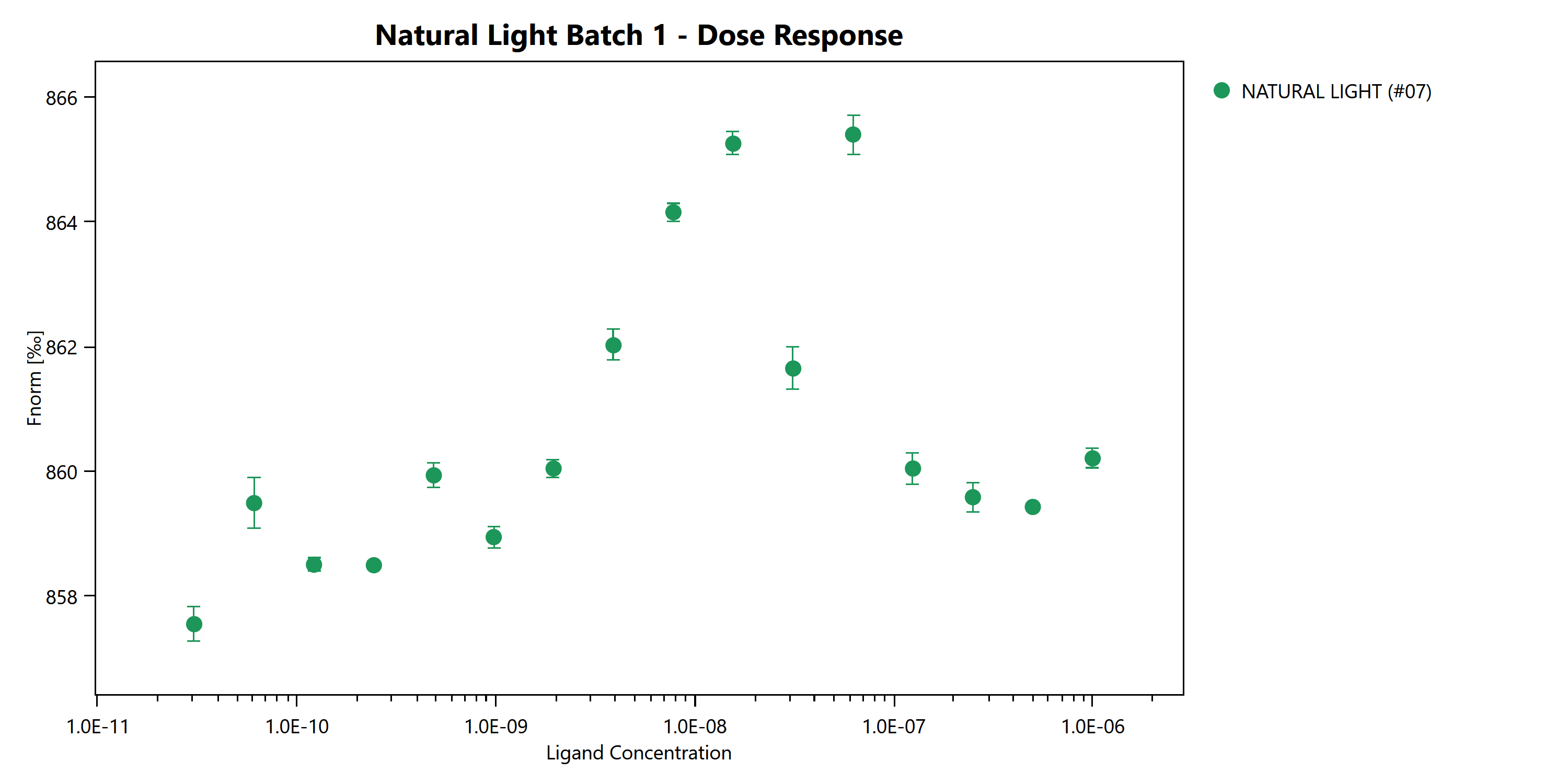 | **B)**  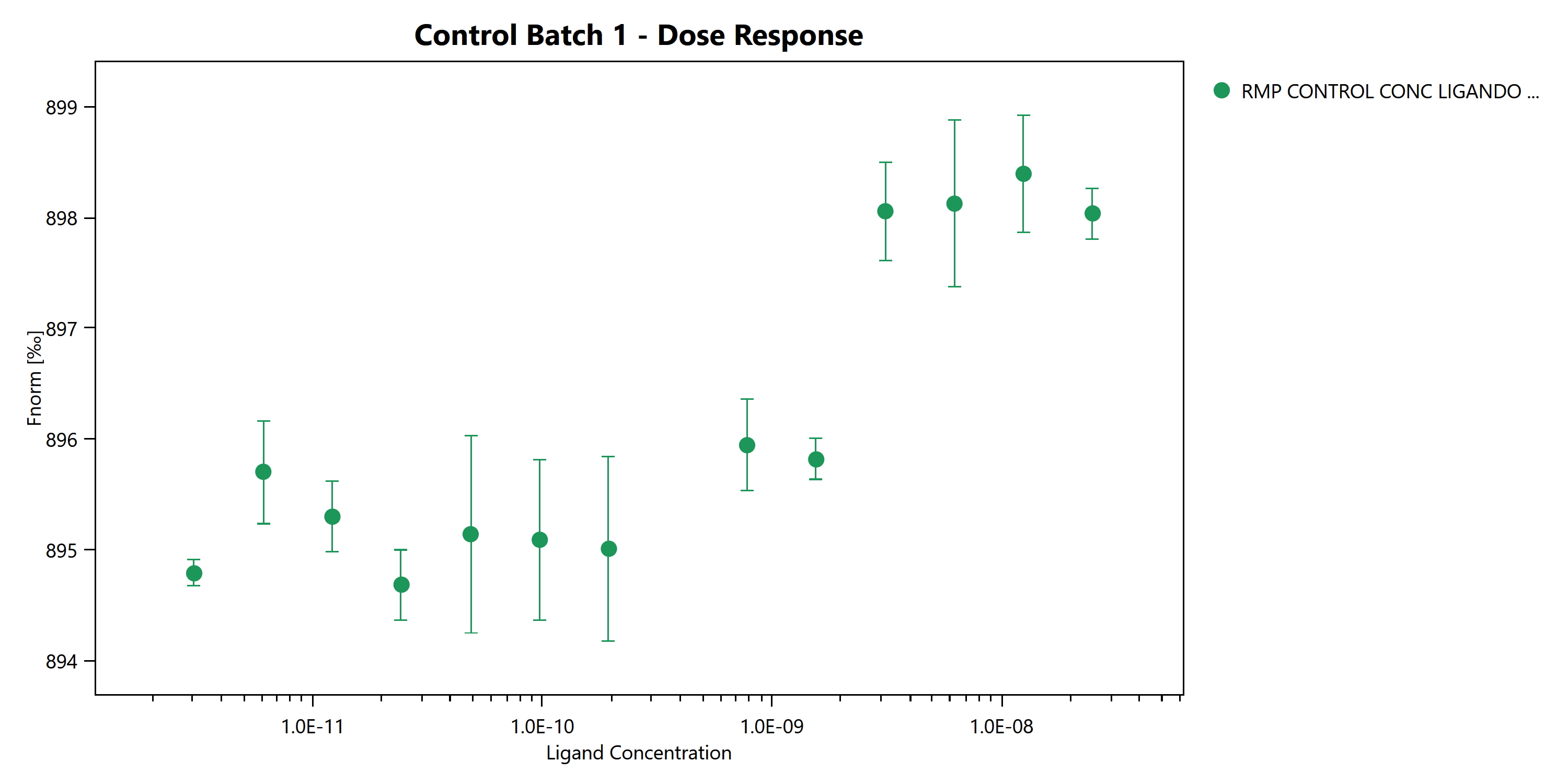 |
| --- | --- |
| **C)**  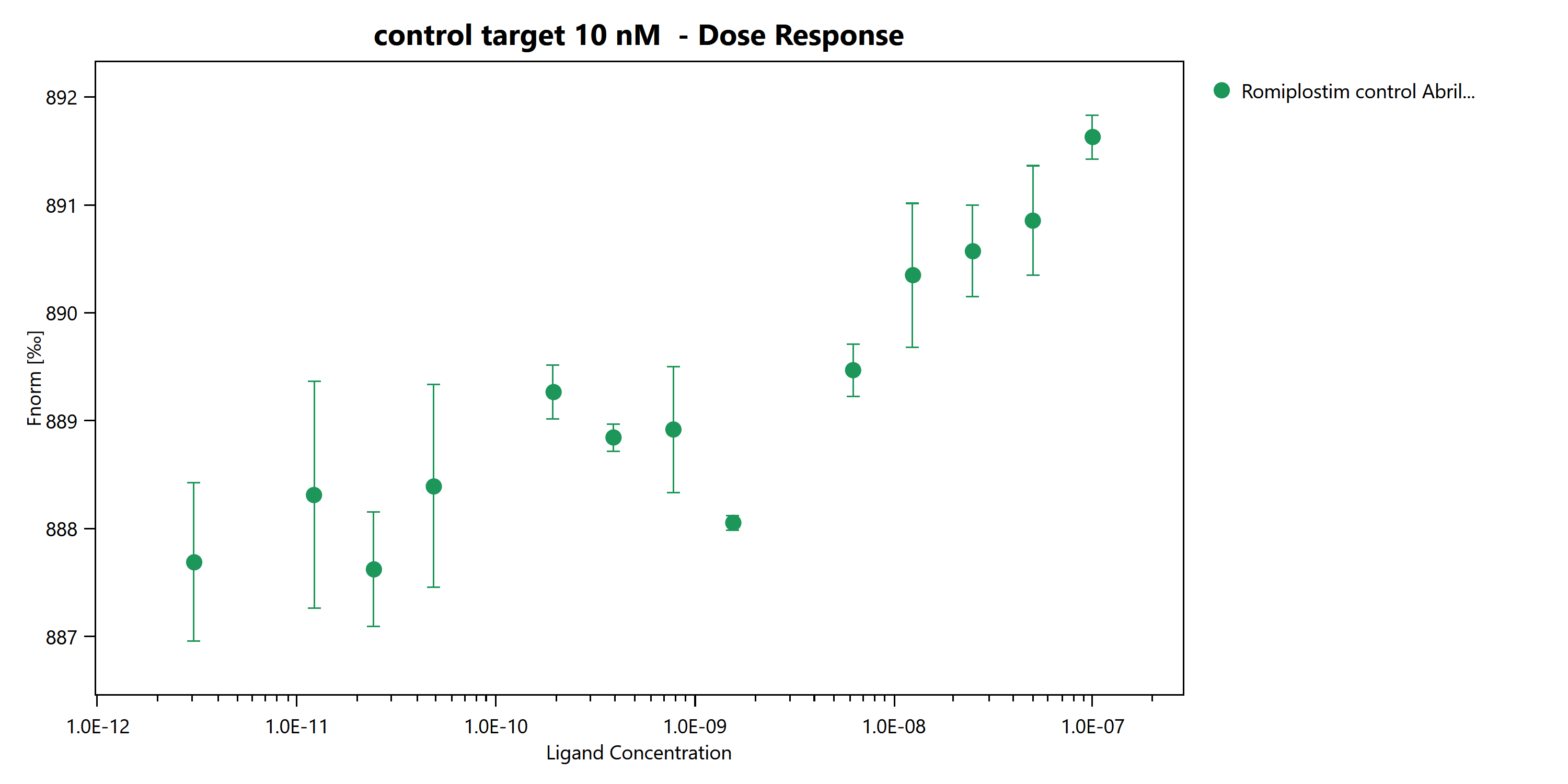 | **D)**  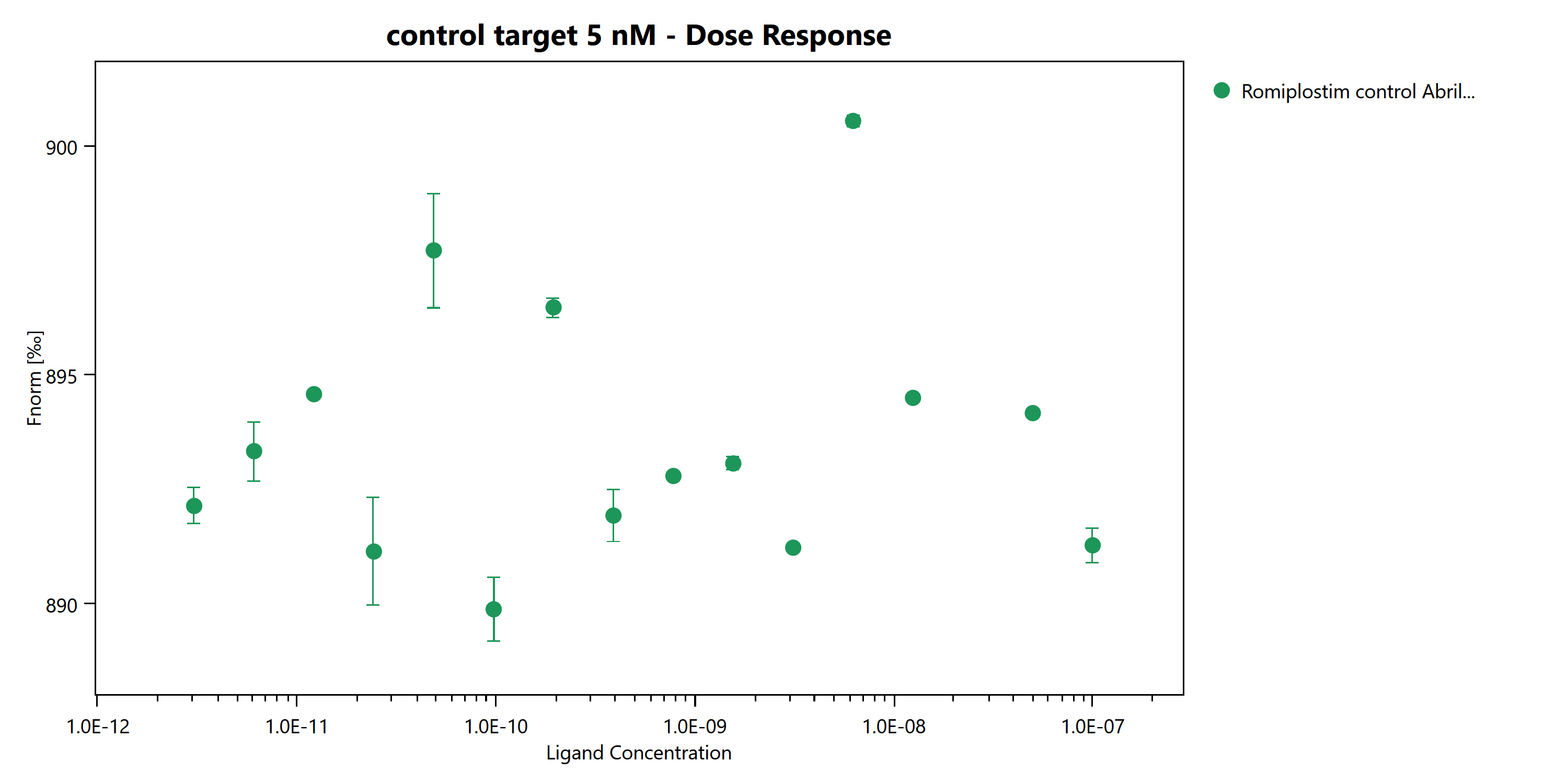 |
| **E)**  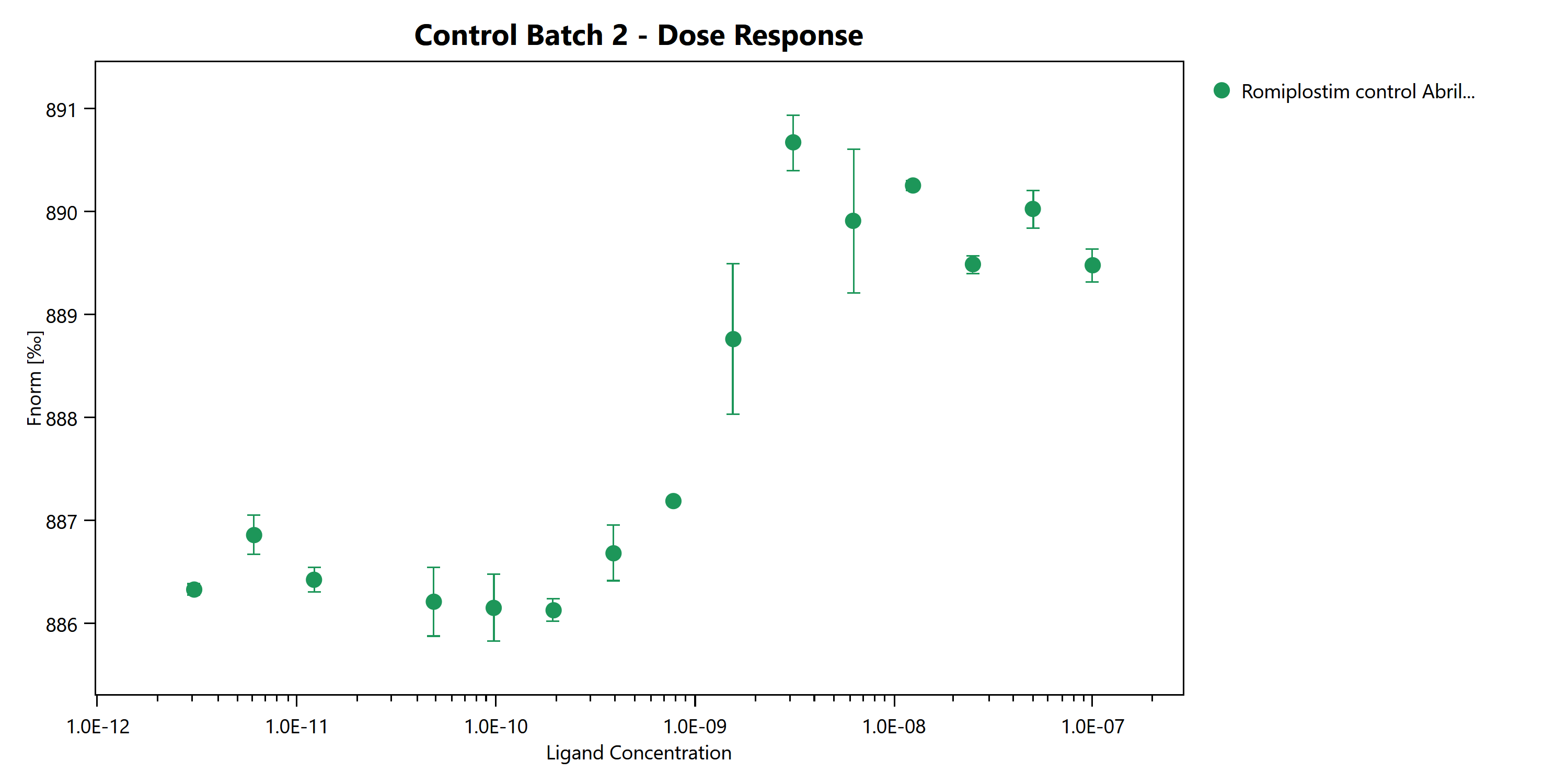 | **F)**  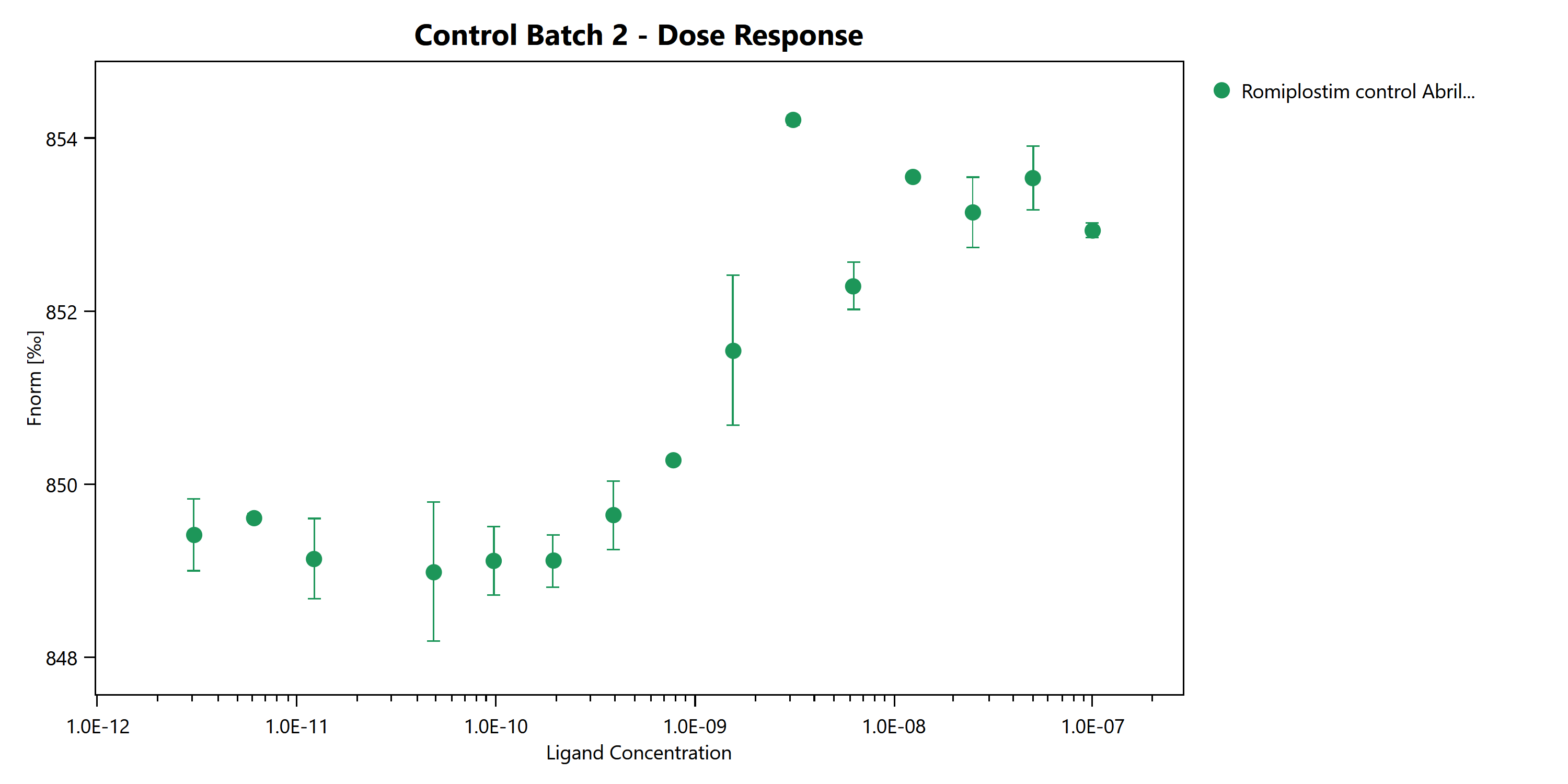 |
| **G)**  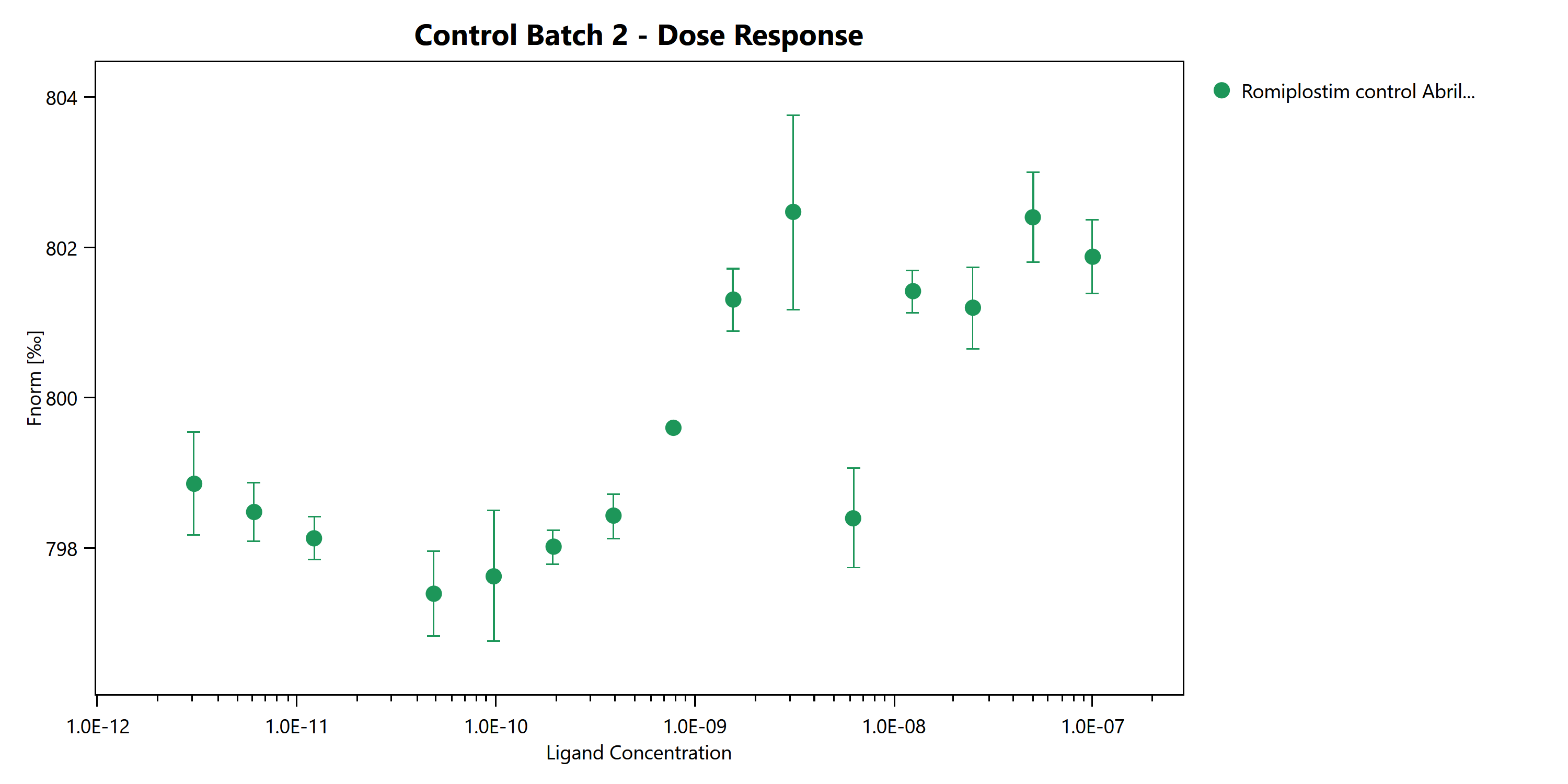 | **H)**  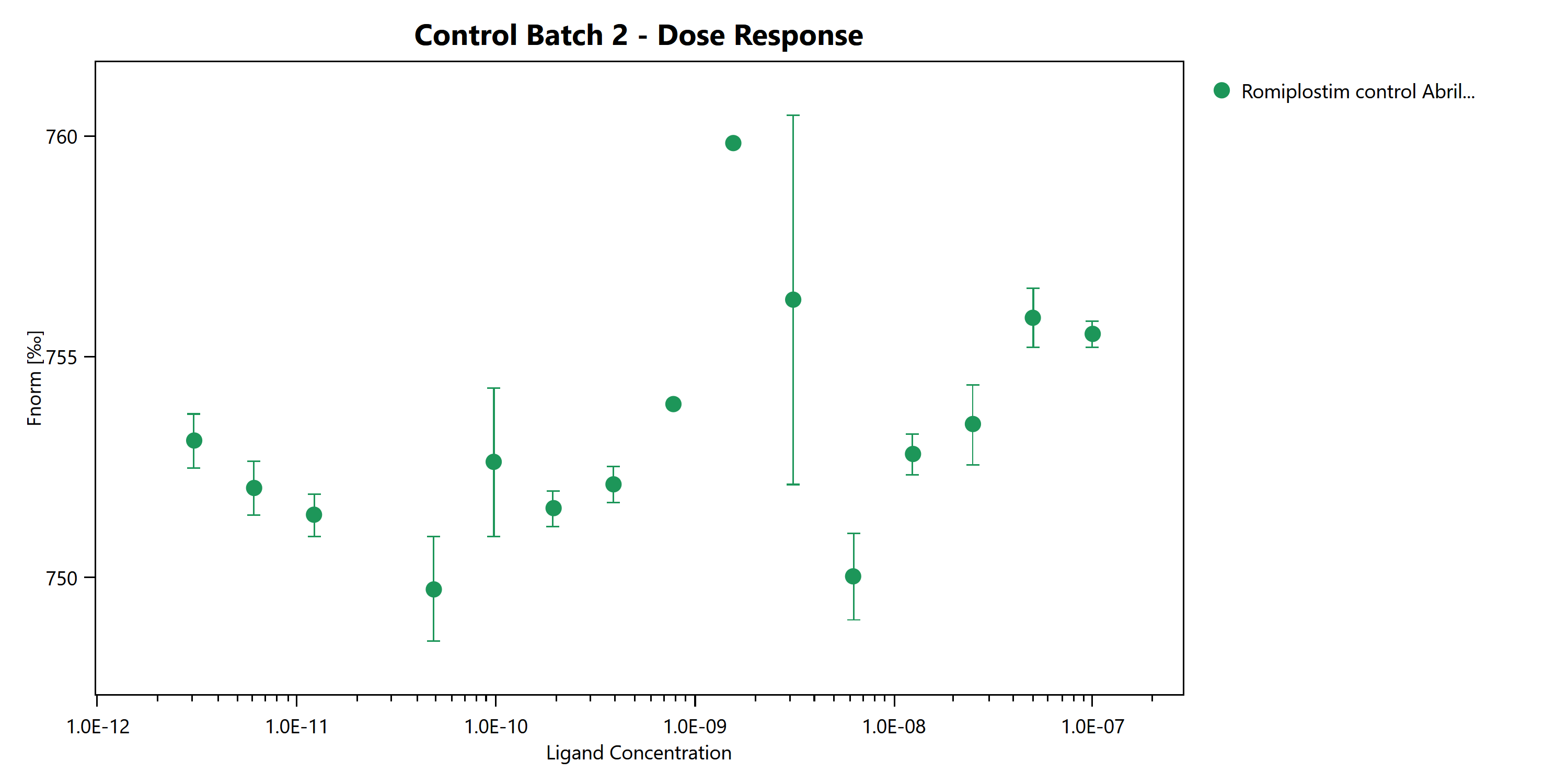 |

**Supplementary Data Figure 3**. MST fluorescence for romiplostim sample subjected to light irradiation stress during 24 h analysed at different times: A) 1.5 s; B) 2.5 s; C) 5 s and D) 10 s. No sigmoidal curve could be observed regardless of analysis time, confirming that no binding occurred in this concentration interval.

| **A)**  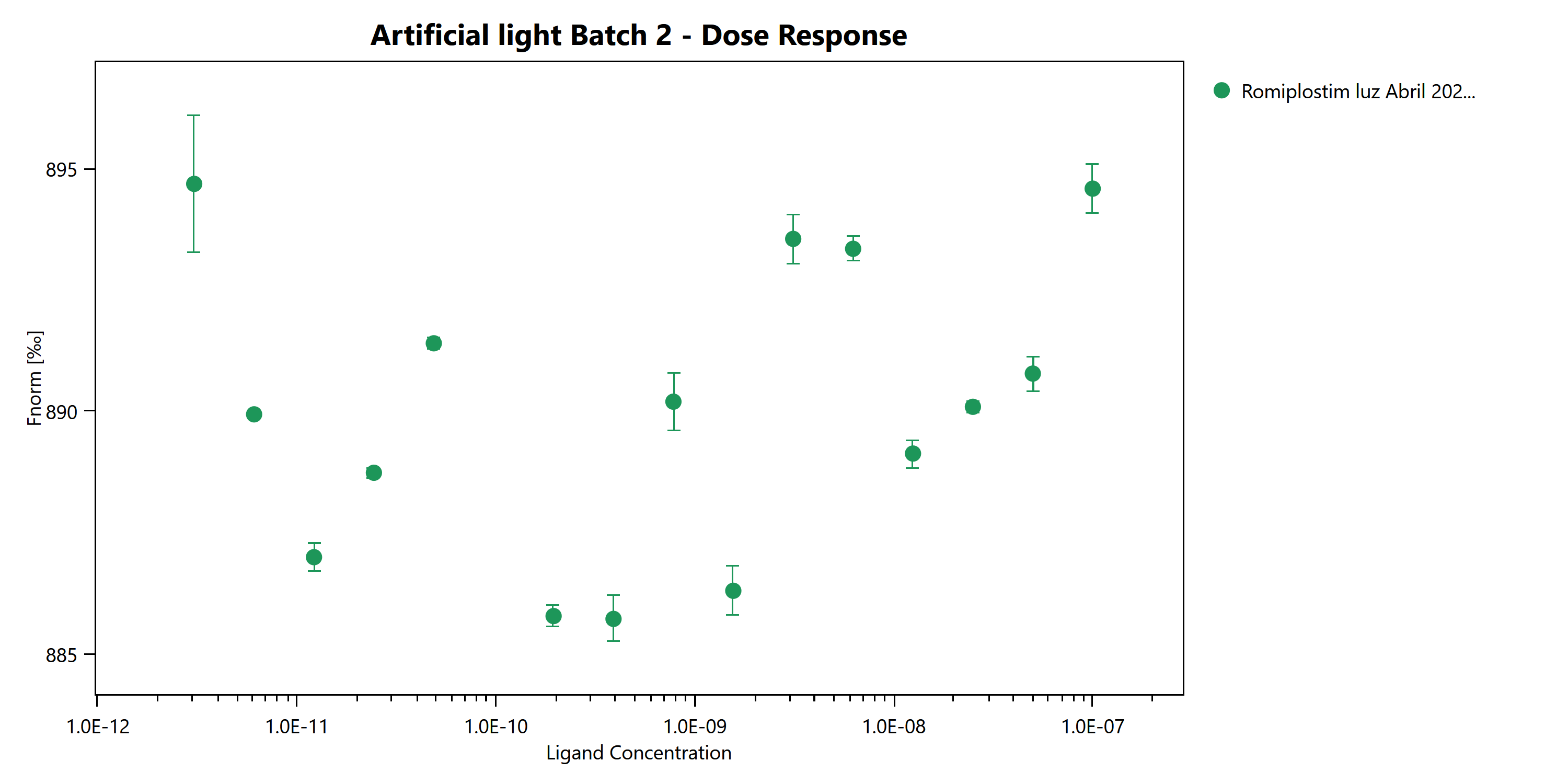 | **B)**  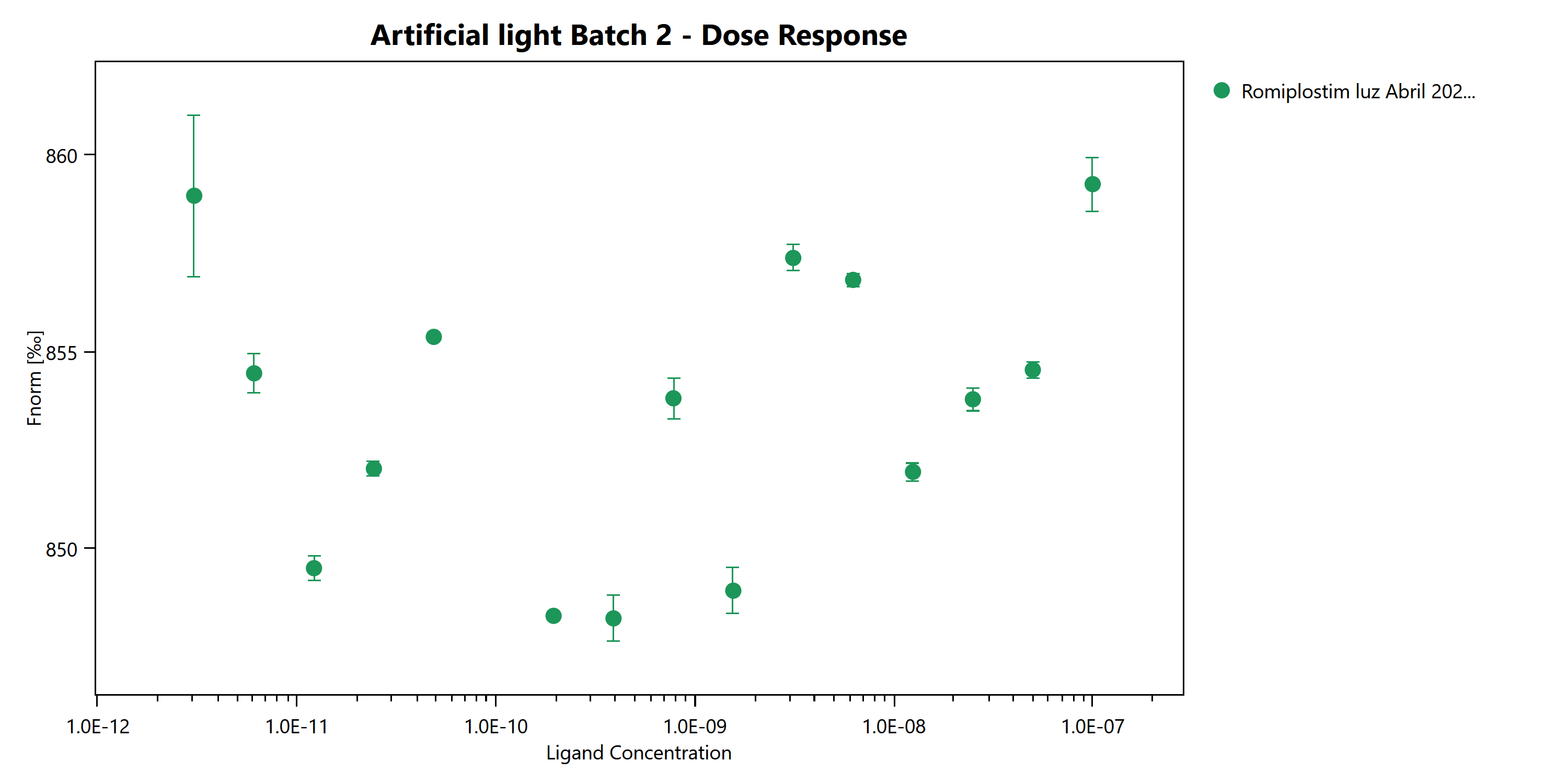 |
| --- | --- |
| **C)**  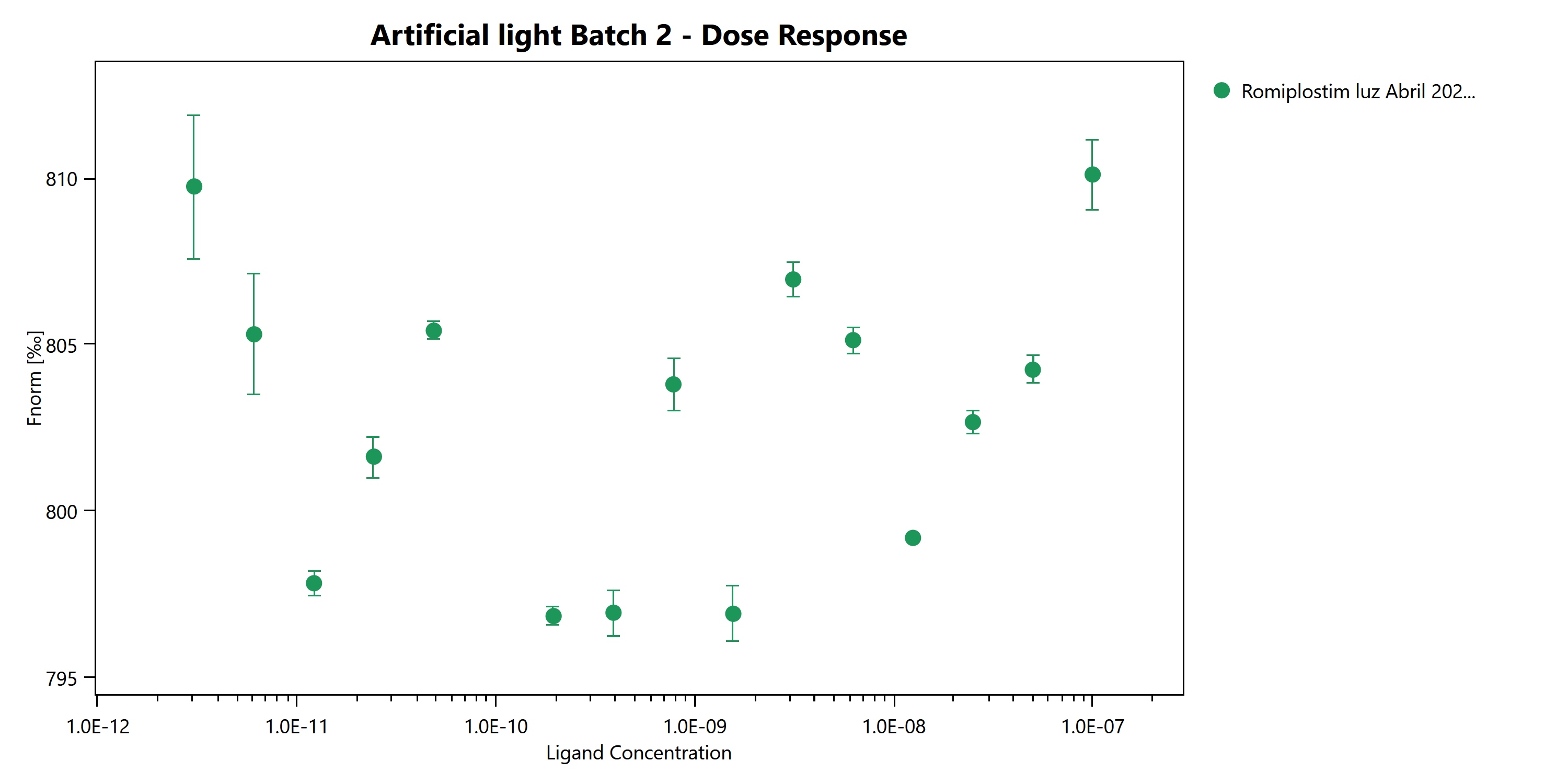 | **D)**  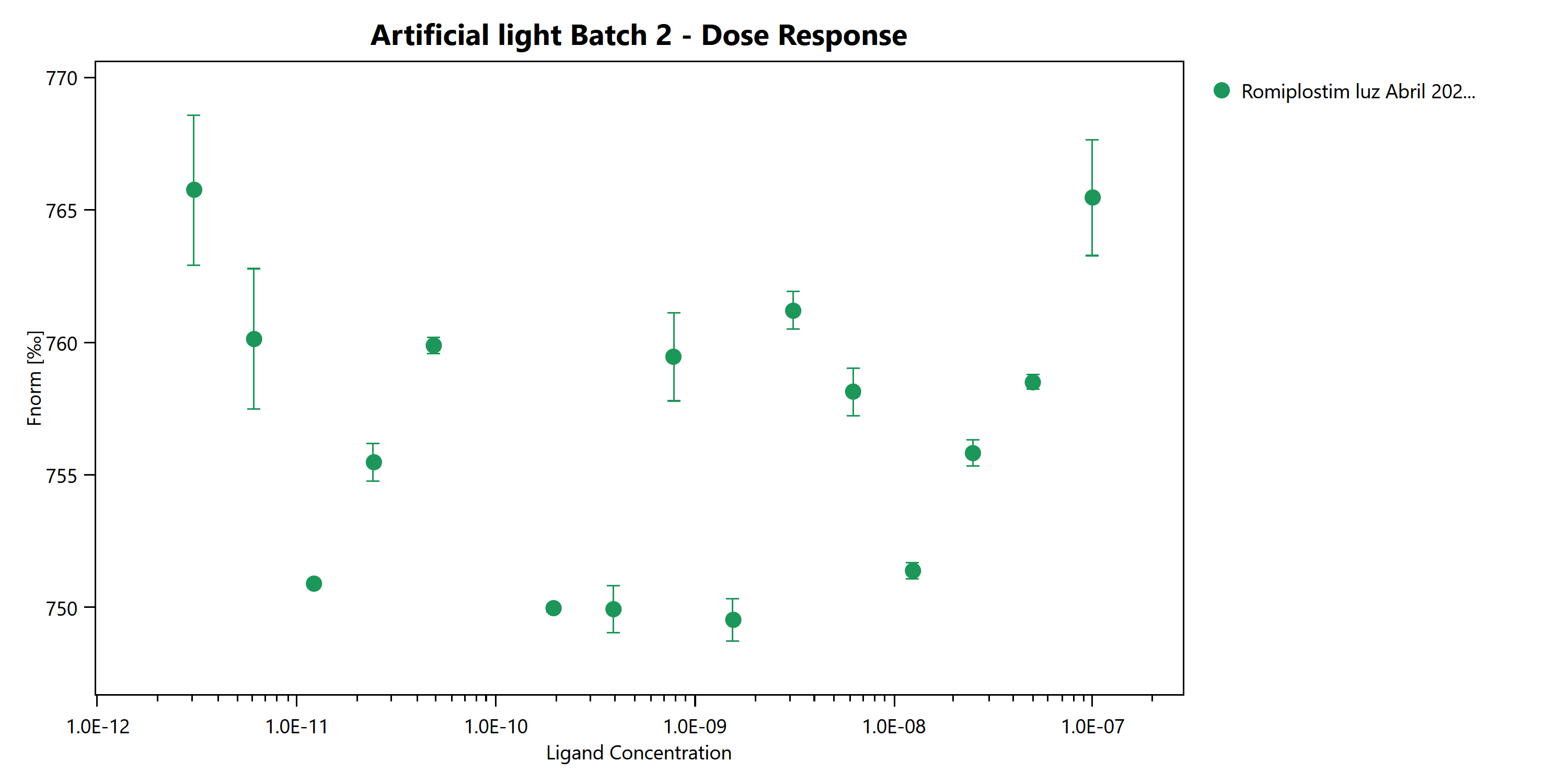 |
